# Lesion of the ventral or dorsal hippocampus in the rat delays puberty, follicular growth and secretion of sex steroid hormones

**DOI:** 10.3389/fendo.2025.1530692

**Published:** 2025-04-16

**Authors:** Estrella del Mar Castillo, Ubaldo Quiróz, Lorena Milflores, Rosalina Reyes, Gonzalo Flores

**Affiliations:** ^1^ Laboratorio de Histofisiología, Facultad de Ciencias Biológicas, Benemérita Universidad Autónoma de Puebla, Puebla, Mexico; ^2^ Laboratorio de Biología Oral, Facultad de Estudios Superiores Zaragoza, Universidad Nacional Autónoma de México (UNAM), Ciudad de Mexico, Mexico; ^3^ Laboratorio de Biología Molecular, Facultad de Ciencias Biológicas, Benemérita Universidad Autónoma de Puebla, Puebla, Mexico; ^4^ Laboratorio de Biología de la Reproducción, Facultad de Ciencias Biológicas, Benemérita Universidad Autónoma de Puebla, Puebla, Mexico; ^5^ Laboratorio de Neuropsiquiatría, Instituto de Fisiología, Benemérita Universidad Autónoma de Puebla, Puebla, Mexico

**Keywords:** hippocampus, NORT, ovary, puberty, follicular growth, steroid hormones, gonadotropins

## Abstract

**Introduction:**

The hypothalamus-pituitary-gonad axis is controlled by gonadotropins and by a direct neural pathway to the gonads. New evidence suggests the existence of neural connection from the hippocampus to the hypothalamus that can regulate its function. It could be a new control on the well-regulated hormonal and neural connection to the gonads and hence in reproduction. The objective of this study was to analyze the effects of independent lesion of the dorsal or ventral hippocampus in the female rat on the onset of puberty, follicular growth and serum concentration of sex steroid and gonadotropins.

**Methods:**

Prepubertal female rats of the CII-ZV strain, 21 days old, were used. Ventral (VH-L) or dorsal (DH-L) hippocampus lesions by the administration of ibotenic acid were performed using stereotaxic surgery. Controls were sham-operated (VH-Sham and DH- Sham), a fifth group was used as absolute control. At 30 days of age all groups underwent novel object recognition tests (NORT).

**Results:**

Data from memory using NORT showed a decrease both in short- and long-term memory in the animals in the VH- L and DH-L groups compared to their respective sham-operated controls and the absolute control group. Similarly, injured rats presented delayed vaginal opening and in first vaginal estrus, a decrease in the number of healthy ovarian follicles and an increase in follicular atresia. The ventral or dorsal hippocampus lesions also caused a significant decrease in the secretion of estradiol and progesterone, an increased plasma testosterone. Only DH-L group showed a significant decrease in serum FSH concentrations compared to their respective control groups.

**Discussion:**

These results show for the first time that the hippocampus participates in a stimulatory manner, that could overcome the gonadotropic control by acting by a neural connection to the gonads giving a novel integrative mechanism between learning processes with neuroendocrine mechanism regulating the ovary function.

## 1 Introduction

Reproduction is an essential process in life that guides biological evolution. It allows each individual to unconsciously compete to perpetuate itself in the next generation. The differential reproductive success of individuals drives the evolution of species (1). The hippocampus is one of the key brain structures involved in learning and memory, which are essential processes in the survival and evolution of species (2–4). Perception of information from outside and inside the organism activates sensory pathways that transport this information to the sensory cortex. The hippocampus receives and processes this information, particularly in the CA3 region (5).

The hippocampus has connections with almost all parts of the limbic system, including especially the amygdala, hippocampal gyrus, cingulate gyrus, hypothalamus and other areas closely related to the hypothalamus. Almost all types of sensory experience originate instantaneous activation in several parts of the hippocampus, which in turn distributes many output signals to the hypothalamus and other parts of the diencephalon via the fornix (6–8). This brain region is divided into dorsal (DH), intermediate (IH) and ventral (VH) hippocampi. While the DH is involved in cognitive functions and its dysfunction leads to amnesia, the HI only separates both regions (9–11). VH is responsible for emotional control, such as frustrations and regulates the activity of the hypothalamic-pituitary-adrenal (HPA) axis (12). It is particularly interesting to consider the role of this ventral portion and that it is strongly linked to circuits related to defensive behavior in the hypothalamus. A clear pattern of stronger projections of the monoaminergic systems to more ventral parts of the hippocampus is also apparent (11), which does not rule out its involvement in reproductive processes.

It is well known that reproduction is regulated by the hypothalamus-pituitary-gonads neuroendocrine axis (HPG). In addition to neuroendocrine regulation, a direct neural pathway has been identified linking the gonads to central nervous system structures, including the hypothalamus and amygdala, which play a critical role in the regulation of gonadal function (13–15). The ovaries receive neural input through noradrenergic, peptidergic, and cholinergic fibers (16–20). In return, they transmit sensory information to the central nervous system via immunoreactive fibers for substance P (SP) and calcitonin gene-related peptide (CGRP) (17–19, 21). Within the dorsal root ganglion, this information is processed by the activation of glutamatergic neurotransmission through both ionotropic and metabotropic receptors (22). In the female rat, denervation of these sensory fibers by capsaicin administration results in delayed puberty, decreased number of ovarian follicles and increased follicular atresia, these changes are related to lower secretion of serum estradiol concentrations (23, 24) and with a significant decrease in the activity of the noradrenergic and serotonergic systems of the anterior hypothalamus (24).

In rodents, sectioning of peripheral sensory nerves causes a decrease in both the number and activity of hippocampal CA1 and CA3 pyramidal neurons (25, 26) and a reconfiguration of neuronal connections between CA3 and CA1 (25). There is evidence that suggests the participation of the hippocampus in the regulation of gonadal functions. The hippocampus has been reported to send information to various hypothalamic regions, which control behavioral, endocrine, and autonomic responses (11). Through the lateral septum, hippocampal information is projected to the median hypothalamus, which is involved in regulating the expression of sexual behavior (27). In Huntington’s disease (HD), neurodegeneration of the hippocampus is observed, which is associated with cognitive dysfunctions, as well as gonadal dysfunction. Interestingly, various studies conducted in humans and animals with HD show changes in sexual behavior, degeneration of energetic GnRH neurons, gonadal atrophy, and alterations in the HPG axis (28, 29). Recently, changes in gene expression in the hippocampus during the estrous cycle have been shown to suggest that variations in sex steroid hormone secretion modify hippocampal GABAergic neural tone (30). Therefore, it allows us to suggest a bidirectional relationship between the hippocampus and the gonads. The objective of this study was to analyze the effects of independent lesion of the dorsal or ventral hippocampus in the female rat on the onset of puberty, follicular growth and serum concentration of sex steroid and gonadotropins.

## 2 Materials and methods

### 2.1 Animals

Twenty-five female neonatal rats of the CII-ZV strain were used, the animals were kept in the “Claude Bernard” biotherium (University of Puebla) under conditions of constant light/dark cycle of 14/10 hrs. and were provided food and water ad libitum. All the procedures described in this study were approved by the Internal Committee for the Care and Use of Laboratory Animals (CICUAL) (100519168-UALVIEP-22/1) and adhered to the ethical framework for Care and Use of Laboratory Animals in accordance with NOM-062-ZOO-1999. Efforts were made to minimize the animals’ suffering and reduce the number of animals used. At 21 days of age, the rats were divided equally into five groups with n=5. Group 1 was lesioned on the ventral hippocampus (VH-L), group 2 lesioned in the dorsal hippocampus (DH-L). Two more groups in the same areas underwent a simulated operation (VH-Sham, DH-Sham) and finally a group of untouched animals (Control).

### 2.2 Ventral and dorsal hippocampal lesion

**Figure 1:**
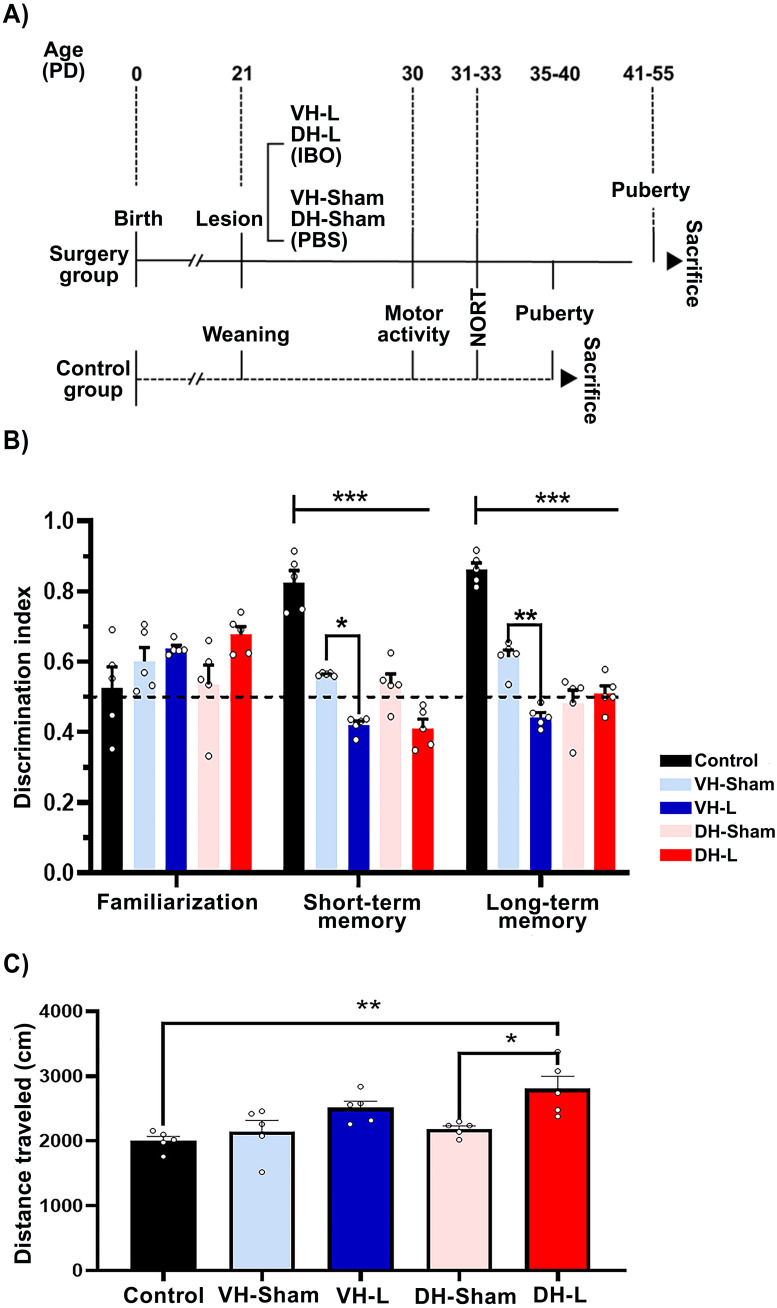
Experimental protocol. **(A)** At PD21 rats in the surgery group (continuous line) were administered PBS bilaterally into the ventral hippocampus to form the sham group, or IBO to form the VH-L and DH-L groups. The intact group (dashed line) was not disturbed until weaning (21 days post-surgery). At PD30–33 days post-surgery, motor activity and NORT were assessed. After reaching puberty, rats were administered an overdose of ketamine-xylazine to obtain samples. **(B)** NORT after lesion at 31 days of postnatal life (short- and long-term memory) of the CII-ZV strain with VH-L, DH-L, sham lesion and untouched animals’ group. The values indicate the recognition or discrimination index (IR) to objects at 0 h, 2h and 24 **(h)** The means ± SEM are presented with n=5 rats per group *p= 0.028, **p= 0.009, ***p< 0.001. **(C)** Motor activity after lesion at 30 days postnatal life of CII-ZV stain. Analysis of the total distance traveled of PD30 rats in open field motor activity post lesion with IBO or PBS. DH-L group traveled farther compared to the sham group and control group, (One-way ANOVA and Bonferroni for *post hoc*; *p = 0.019, **p= 0.002).

The VH-L and DH-L protocol was performed according to previous reports (31, 32). Briefly, considering the day of birth as postnatal day (PD) 0 at PD21, the female offspring (n=20) were anesthetized by ketamine-xylazine and fixed to a neonatal adapter (33) attached to a stereotaxic frame. Once the ventral and dorsal coordinates of the hippocampus were located (VH: anteroposterior: -5.2 mm, medial-lateral: +/- 5.0 mm, both from Bregma; and dorso-ventral: -6.5 mm from dura mater; DH: anteroposterior: -4.2 mm, medial-lateral: +/- 2.6 mm, both from Bregma; and dorso-ventral: -3.0 mm) 0.3 uL of ibotenic acid (IBO) (10 ug/uL; Sigma, St. Louis, MO) for animals with VH-L and DH-L (n=10) or the same vehicle volume (0,1 M phosphate-buffered saline [PBS], pH 7,4) for sham rats (n=10) were administered bilaterally during 3 minutes to ensure a proper administration, then the animals were sutured and administrated using a dose of the antibiotic enrofloxacin and the analgesic ketoprofen (0.20 ml/100 g live weight s.c.) and were left to recover under the indirect heat of a lamp to regain their temperature. For the intact group (n=5), the offspring were not disturbed until reaching puberty. The diagram representing the experimental protocol is shown in **Figure 1A**.

### 2.3 Behavior tests

At prepuberal age, the behavior of the animals was evaluated. Locomotor activity was estimated at PD30. Then, from PD31 to PD33, the novel object recognition test (NORT) was performed.

### 2.4 Locomotor activity

An open field test was carried out for these ages (n = 5 per group), rats were kept thirty minutes before test in the work area with red lighting for their adaptation. This test began placing the animal on square 7 of a wooden box (60x60x60 cm) previously cleaned and divided into nine squares of 20x20 cm each for 10 minutes, the observer registered the number of squares visited considering as a visit when all four paws of the animal were inside the square (34–36) After the test, the animal was removed, and the corresponding cleaning was carried out to avoid influencing the conduct of the rodents by animal’s odor.

### 2.5 Novel object recognition test

To evaluate the memory performance of rats, the NORT was employed as previously reported with some modifications (36–38). The test lasted three days and was assessed between 7:00–12:00 h, in a dark room with red light (30 lx) and isolated noise. The first day, the rats explored an open field box (60 x 60 x 60 cm) during 10 min (habituation stage). On the second and third day, the animals explored two objects located in the box for 6 min as described below. The second day, 24 hrs. after the habituation stage, the rats explored two objects that had the same characteristics (familiarization stage). 2 hrs. after the familiarization stage, an object was replaced by a novel one and the other object remained without change (short term retention stage). Finally, on the third day, 24 hrs. after the short-term retention stage, the novel object that had previously been changed was replaced by a new one and the other object remained unchanged. (long term retention stage). For each rat, the amount of exploration time with the new object was counted. The recognition index (IR) was determined with the following equation: *I_R_* = *T_N/_* (*T_K_* +*T_N_*). Where *I_R_* is the recognition index, *T_N_* is the time that each animal explores the new object and *T_K_* is the time that each animal explores the known object.

### 2.6 Evaluation of the estrous cycle

The vaginal smears were collected between 09:00 and 10:00 hrs. daily, after the vaginal opening. The smears were identified by microscopic examination of the cell types and were classified into 1 of 4 phases of the estrous cycle: proestrus, estrus, metestrus, or diestrus. The phases of the estrous cycle were identified according to the predominant cell type observed in the vaginal smears when examined with an optical microscope (39). Only rats that were shown to be in the estrus phase were sacrificed. Upon reaching puberty, the rats were anesthetized with an overdose of ketamine-xylazine (0.20 ml/100 g live weight i.p.) and perfused intracardially with 0.9% NaCl. Immediately at least 3 ml of blood was collected by cardiac puncture and the sample was centrifuged at 3000 rpm for 15 minutes to obtain the serum subsequent evaluations of estradiol, progesterone, testosterone, FSH and LH concentrations, as described below.

### 2.7 ELISA for measuring hormone concentrations

ELISA was used to measure estradiol (pg/ml), progesterone (ng/ml), testosterone (ng/ml), FSH (mlU/ml) and LH (mlU/ml) serum levels, using reagents and protocol supplied by MEXLAB (México). The absorbance of the reaction of all hormones was measured at a wavelength of 450 nm in a spectrophotometer. The intra- and inter assay percent variation coefficients for estradiol were 6.3 and 6.8, progesterone 5.3 and 9.68, testosterone 5.76 and 6.87, FSH 6.3 and 6.8 and for LH 6.18 and 8.13, respectively.

### 2.8 Histology

#### 2.8.1 Golgi-Cox staining method

For the morphological parameters of the dendritic spines, the Golgi-Cox staining method was used with some modifications (40). These staining methods were performed as previously described (41–43). Once the animals were sacrificed the brains were removed from the skull, immersed in Golgi-Cox solution for 30 days in the dark. After that, 200 μm thick coronal sections from the regions of interest were obtained using a vibratome (Campden Instruments, Leicester, UK) and collected on gelatin-coated microscope slides and immersed in ammonium hydroxide (30 min), then in Kodak Rapid Fixer (30 min). Subsequently, the slides were rinsed in distilled water, dehydrated with sequential increasing ethanol concentration baths (50% for 1 min, 75% for 1 min, 90% for 1 min and 100% twice for 5 min), followed by rinsing in xylene solution (15 minutes). Finally, the slides were mounted with resinous microscopy medium.

#### 2.8.2 Number of dendritic spines and their morphology

The basal dendritic tree of the pyramidal neurons of the CA1, CA3, DG regions of the hippocampus was located, the stereotaxic atlas of (44) was used as a guide. For each rat, five dendritic segments were reproduced (a length 5.4 cm equivalent to 30 μm, of the most distal dendrite of the neurons in each hemisphere of the hippocampal areas using a lucid camera with a 1000x magnification (Leica DMLS microscope) to estimate the density of the dendritic spines On the same dendritic segments, the characterization of the spines according to their shape was performed as previously described with some modifications (41). For this purpose, using a 2x lens with a 2000x magnification (Leica DMLS microscope), one hundred spines were classified according to the shape of their head and neck into five groups: mushroom spines (prominent head with well identified neck), thin spines (elongated spines with head/neck of similar thickness), thick spines (wide spines with an indistinguishable neck), branched spines (spines with two heads), and unclassified spines (inconsistent with any of above criteria). A trained observer who was unaware of the experimental conditions performed the morphological and density classification of the spines.

#### 2.8.3 Ovarian staining

The ovaries were fixed in Bouin solution, embedded in paraffin wax, serially sectioned at a thickness of 10 μm and stained with hematoxylin-eosin. All ovarian sections were analyzed with an optical microscope. (Primo Star microscope, ZEISS, Jena, Germany).

### 2.9 Follicular growth analysis

To determine the follicular growth, the number of follicles with presence (antral) or absence (preantral) of the follicular antrum was quantified. Those follicles that had a fluid filled space and several layers of granulosa cells were classified as antral follicles; those with only one layer of flattened pregranulosa cells and one of two layers of cubic granulosa cells were considered preantral follicles. For this evaluation, both healthy follicles and those showing signs of atresia were included for the analysis.

### 2.10 Follicular maturation analysis

Follicular maturation is calculated by quantifying follicle types (45), considering the following parameters: Primordial: primary oocyte surrounded by a single layer of flattened pregranulosa cells. Primary: primary oocyte surrounded by a single layer of cubic granulosa cells. Secondary: primary oocyte surrounded by more than one layer of granulosa cells and with antral formation (fluid filled space). Tertiary: (also named and known as ovulatory follicle): late stage (or large) antral follicle (approximately 700 μm in diameter) where the primary oocyte is surrounded by an oophorus group, and the follicle will respond to the sudden increases in LH level.

### 2.11 Analysis of the follicular status

The number of healthy and atretic follicles was quantified. Considering signs of follicular atresia: n nuclear pyknosis of granulosa cells, theca thickening, desquamation and oocyte fragmentation were analyzed.

### 2.12 Statistical analysis

All the data are presented as the mean ± standard error of the mean (SEM). A normality test was performed to verify that all sample distributions were normal and had equal variances. For the data analysis, a one-way ANOVA and two-way ANOVA were performed with the lesion and the testing phase as independent variables with a *post-hoc* uncorrected Bonferroni test for multiple comparisons using the GraphPad Prism 8 software (GraphPad Software, CA, USA). Values of P<0.05 were considered significant.

## 3 Results

### 3.1 Behavioral testing

Behavioral tests in a novel environment were employed to assess short-term and long-term memory, as well as the effects of VH-L, DH-L, and sham lesions in rats corresponding to each region, along with an intact control group, through the novel object recognition task.

### 3.2 Novel object recognition after lesion in 31-day old rats

The behavioral testing of NORT in CII-ZV strain rats were performed after the lesion, at 31 days postnatal life with a recognition index (IR) that evaluates the time spent with a novel object versus a familiar object, a two-way ANOVA was performed with F (8, 60) =7.650, p<0.001 for the lesion phase followed by Turkey´s test p<0.05. It is observed that in the training phase the recognition indices of both groups are close to 0.5, this suggests that the groups do not present any preference for any of the objects. After two hours of the first exposure phase, a decrease in the recognition index of the experimental groups (VH-L, VH-Sham, DH-L, DH-Sham) can be observed in comparison with the control group, and lastly at 24 hours after the last exposure, a decrease in the recognition index is shown in the same experimental groups. On the other hand, the VH-L group presents a decreased recognition index for both short term and long-term memory phase compared to its VH-Sham control group (**Figure 1B**). This was performed using a two-way ANOVA: ***p< 0.001, **p= 0.009, *p= 0.028.

The effect of lesions on the ventral hippocampus (VH-L) and dorsal hippocampus (DH-L) were evaluated. The analysis shows that dorsal hippocampal (DH-L) lesioned rats have a higher motor activity F (4, 20) = 6.492, p =0.0016) compared to the untouched animals (Control) ** p = 0.0020 and their control group (DH-Sham) * p =0.0117, one-way ANOVA and Tukey’s test for multiple comparisons were performed (**Figure 1C**).

### 3.3 Puberty parameters

**Figure 2:**
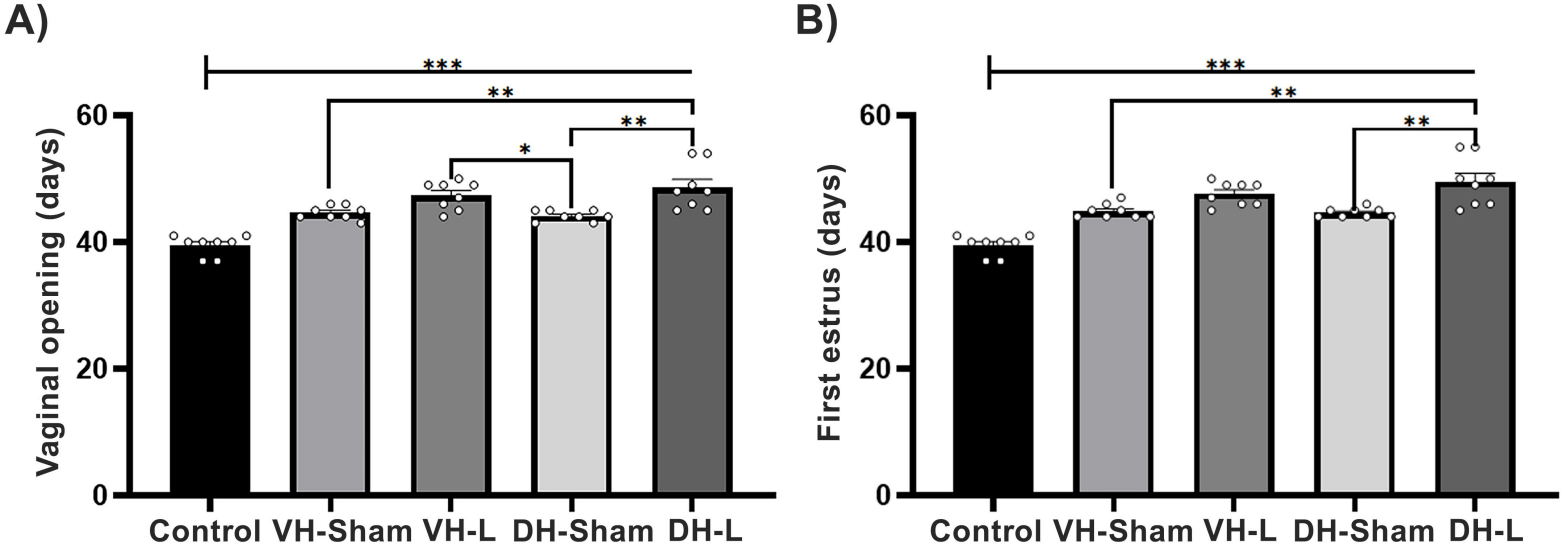
Parameters of puberty. **(A)** Comparison of the age at vaginal opening and **(B)** first estrus in pubertal female rats with dorsal (DH-L) and ventral (VH-L) hippocampal lesions, sham lesions, and an untouched control group of the CII-ZV strain. The VH-L and DH-L groups exhibited a significant delay in puberty onset compared to the sham and control groups (One-way ANOVA followed by Tukey’s *post hoc* test; ***p < 0.0001, **p < 0.001, *p < 0.03).

The registry of the ages of the vaginal opening and first estrus of injured animals at 21 days of age in dorsal or ventral hippocampus and a control group showed significant differences (Vaginal opening: F (4, 35) = 22.29 p<0.0001; First estrus: F (4, 35) = 24.97 p<0.0001.The ibotenic acid lesioning performed in the dorsal and ventral hippocampal regions in 21-day postnatal rats resulted in delayed age at vaginal opening (VH-L: 47.38 ± 0.77 vs VH-Sham: 44.63 ± 0.37 vs Control group: 39.50 ± 0.56; DH-L: 48.63 ± 1.28 vs DH-Sham: 44.13 ± 0.29 vs Control group: 39.50 ± 0.56); compared to rats with only mechanical injury and in animals without any damage (**Figure 2A**). Upon analyzing the age at first estrus, significant differences were presented in all experimental groups (VH-L: 47.63 ± 0.65 vs VH-Sham: 44.88 ± 0.39 vs Control group: 39.50 ± 0.56; DH-L: 49.50 ± 1.37 vs DH-Sham: 44.63 ± 0.26 vs Control group: 39.50 ± 0.56) (**Figure 2B**).

### 3.4 Ovarian morphology (Follicular population)

**Figure 3:**
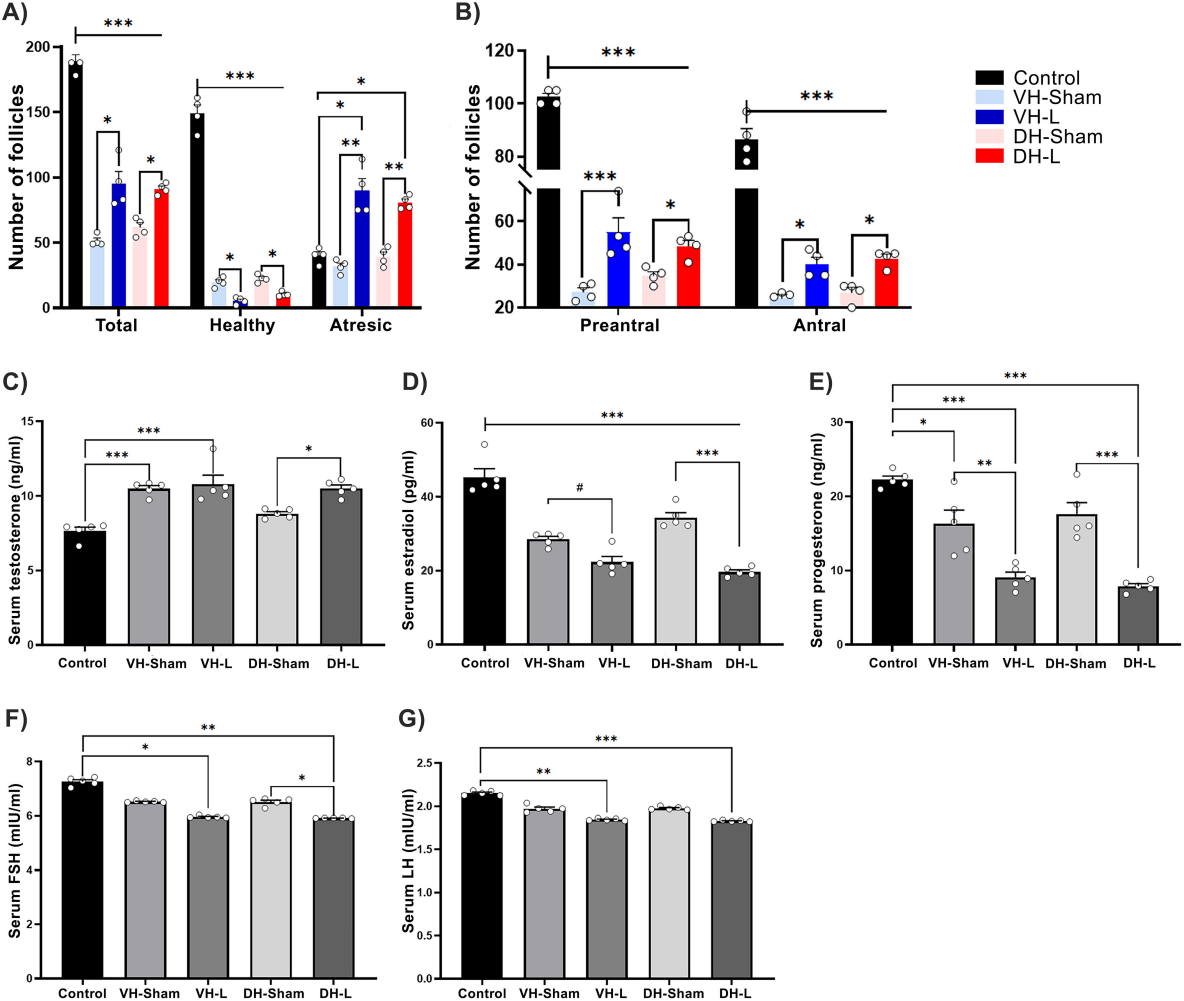
Effects of hippocampal lesion on the HPG axis. **(A)** Lesions induced in the ventral (VH-L) and dorsal (DH-L) hippocampus resulted in a decrease in healthy follicles and an increase in atretic follicles compared to the sham groups (*p = 0.03, ***p = 0.0007). **(B)** The VH-L and DH-L groups exhibited a significant reduction in preantral and antral follicles compared to the control group, while showing an increase relative to the sham groups. Data are presented as mean ± SEM, with n= 4 rats per group (two-way ANOVA followed by Tukey’s *post hoc* test; *p = 0.01, ***p < 0.0001). **(C)** VH-L and DH-L increase serum testosterone secretion relative to their control groups. **(D)** The lesions performed in ventral and dorsal hippocampus cause a decrease in serum estradiol release. **(E)** The serum progesterone release was significantly decreased in the ventral and dorsal hippocampal lesion groups. Statistics were performed by one way ANOVA test, Tukey’s test for multiple comparisons; *p = 0.0143, #p =0.0121, **p = 0.0020, ***p < 0.001. **(F)** VH-L and DH-L decrease serum FSH secretion. **(G)** Shows the decrease in serum LH concentration in animals with ventral and dorsal hippocampal lesion. (One-way ANOVA, Tukey’s test for multiple comparisons; *p = 0.0127, #p = 0.0399, **p = 0.0074, ***p =0.0003.

When analyzing the follicular population, the results showed a decrease in the number of healthy follicles and an increase in atretic follicles in the experimental groups compared to the VH-Sham, DH-Sham, and the Control group (F (1.733, 5.198) = 120.3) (**Figure 3A**). Two-way ANOVA, Tukey’s test for multiple comparisons.

The classification of follicles in preantral and antral, shows that the ventral or dorsal hippocampal lesion causes a significant decrease in both types of follicles observed compared to the control group, two-way ANOVA, turkey tests for multiple comparisons: *p=0.01, ***p<0.0001 (**Figure 3B**).

### 3.5 Effect of lesion on steroid hormone secretion in CII-ZV rats

The ibotenic acid lesion in ventral and dorsal hippocampal areas significantly increased serum testosterone concentration (**Figure 3C**), (F (4, 20) = 136.5, p=0.0001), p < 0.0001 vs. control, p < 0.0001 vs. sham lesion). Regarding serum estradiol concentrations, a decrease was observed (**Figure 3D**), (F(4, 20) = 52.71, p < 0.0001), p < 0.0001 VH-L, DH-L vs Control group, p=0.0413 VH L vs VH L Sham and p < 0.0001 DH-L vs DH-Sham) as for serum progesterone results a significant decrease was also observed (**Figure 3E**), (F(4, 20) = 27.85, p < 0.0001), p < 0.0001 VH-L, DH-L vs control group, p=0.0020 VH-L vs VH-Sham and p < 0.0001 DH-L vs DH-Sham.

### 3.6 Effect of lesion on gonadotropin secretion in CII-ZV rats

The lesions performed in the ventral and dorsal hippocampal areas significantly decreased serum FSH concentration (**Figure 3F**), (F (4, 20) = 164.7, p <0.0001), p =0.0127 VH-L vs Control group, p =0.0074: DH-L vs Control group, p= 0.399 DH-L vs DH-Sham. Regarding serum LH concentrations, the lesions performed in the VH-L and DH-L groups caused a decrease in said hormone, compared to the control group. (**Figure 3G**), (F(4, 20) = 136.5, p < 0.0001), p = 0.0074 VH-L vs Control group, p <0.0003: DH-L vs Control group.

### 3.7 Effect of lesions on structural neuroplasticity in hippocampal regions of CII-ZV rats

**Figure 4:**
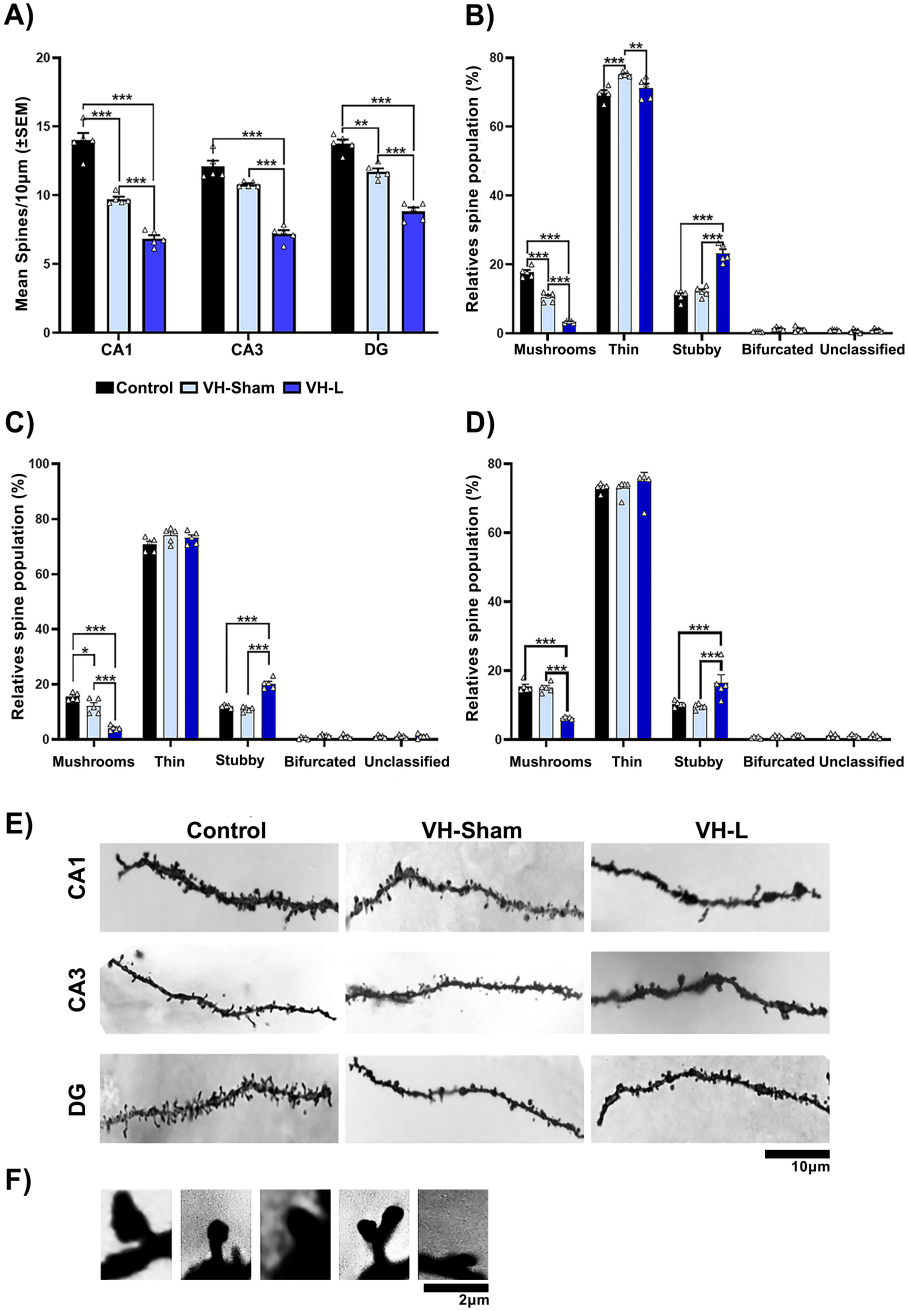
Structural neuroplasticity of the dorsal hippocampus of CII-ZV rats with VH-L. **(A)** VH-L decreases the number of spines in these neurons (CA1, CA3 and DG). **(B-D)** Morphological classification of dendritic spines. **(B)** number of spines in the CA1 region. VH-L reduces the number of mushrooms and thin spines and increases the number of thick spines. **(C, D)**, Number of dendritic spines in CA3 and DG region, VH-L decreases the number of fungal spines and increases the number of thick spines (two-way ANOVA, Bonferroni test for multiple comparisons: *p = 0.033, **p = 0.005, ***p < 0.001. **(E)** Dendritic spine density and morphology. Representative photomicrographs of distal segments of the basal dendritic arbor of pyramidal neurons from CA1, CA3, and DG regions of the dorsal hippocampus from each of the groups, (n=5 per group; scale bar = 10 μm). **(F)** Representative photomicrographs of each type of spine, (scale bar=2 μm).

**Figure 5:**
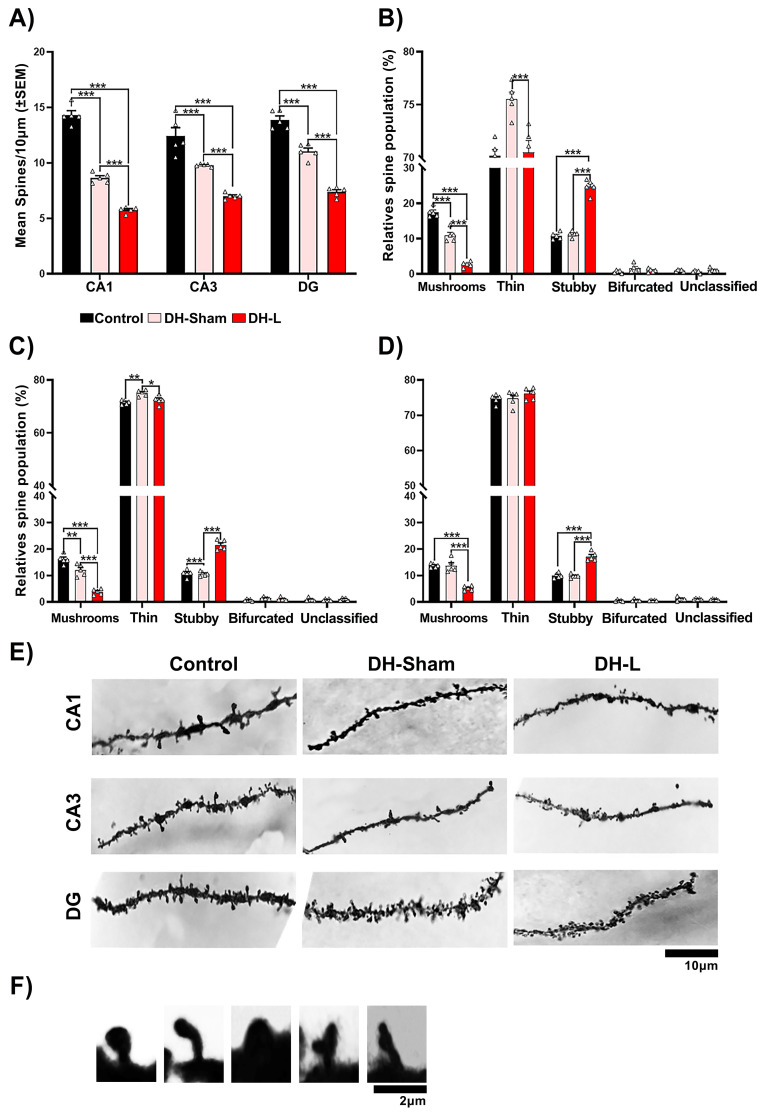
Structural neuroplasticity of the ventral hippocampus of CII-ZV rats with DH-L. **(A)** DH Lesion decreases the number of spines in these neurons (CA1, CA3 and DG). **(B-D)** Morphological classification of dendritic spines. **(B)** number of spines in the CA1 region. DH Lesion reduces the number of mushroom and thin spines and increases the number of thick spines. **(C, D)**, Number of dendritic spines in CA3 and DG region, DH Lesion decreases the number of fungal spines and increases the number of thick spines percentages of these spine types (two-way ANOVA, Bonferroni test for multiple comparisons: *p= 0.02, ***p = 0.001. **(E)** Dendritic spine density and morphology. Representative photomicrographs of distal segments of the basal dendritic arbor of pyramidal neurons from CA1, CA3, and DG regions of the ventral hippocampus from each of the groups, (n=5 per group; scale bar = 10 μm). **(F)** Representative photomicrographs of each type of spine, (scale bar=2 μm).

Rats with VH-L and DH-L had severe alterations in the dendritic spine density and dynamics in the region CA1, CA3, DG the hippocampus (**Figures 4E, F**, **5E, F**). In the dorsal hippocampal area (**Figure 4A**), the ventral hippocampal lesion (VH-L) reduced the density of basilar dendritic spines of pyramidal and granular cells (F(2, 8) = 33.69, p=0.0001), p<0.0001 vs. control, p<0.0001 vs. sham lesion), in all three regions evaluated. Regarding the morphological classification of dendritic spines VH-L reduced the number of mushroom spines in the CA1 region (**Figure 4B**), (F(2, 12) = 184.2, p < 0.0001; p < 0.0001 vs. Control and Sham lesion) and thin spines (F(2, 12) = 8.960, p=0.0042; p=0.005 vs. Sham lesion), but increased the number of thick spines (F(2, 12) = 59.15, p<0.0001; p<0.0001 vs. control, p<0.001 vs. sham lesion). The VH-L had no effect on the number of bifurcated and unsorted spines of these neurons.

Morphological classification of dendritic spines VH L reduced the number of fungal spines in the CA3 region (**Figure 4C**), (F(2, 12) = 51.61, p<0.0001; p<0.0001 vs. Control and Sham lesion) and of thin spines no significant differences were observed (F(2, 12) = 2.295, p=0.1432), but it did increase the number thick spines (F(2, 12) = 86.26, p < 0.0001; p <0.0001 vs. Control and Sham lesion). The VH-L also had no effect on the number of bifurcated and unsorted spines of these neurons.

Lastly, in the DG region there was also a decrease in mushroom like spines (**Figure 4D**) (F(2, 12) = 75.32, p <0.0001; p <0.0001 vs. Control and Sham lesion) and thin spines no significant differences were observed, but the number of thick spines increased (F(2, 12) = 8.196, p = 0.0057; p =0.0005 vs. control and p =0.001 sham lesion). The VH-L had no effect on the number of bifurcated and unsorted spines of these neurons.

In the ventral hippocampal area (**Figure 5A**), the dorsal hippocampal lesion (DH-L) reduced the density of basilar dendritic spines of pyramidal cells (F(2, 8) = 25.95, p=0.0003), p < 0.0001 vs. Control, p < 0.0001 vs. Sham lesion), in all three hippocampal regions evaluated. Regarding the morphological classification of dendritic spines the DH-L reduced the number of mushrooms spines in CA1 region (**Figure 5B**), (F(2, 12) = 139.2, p < 0.0001; p < 0.0001 vs. Control and Sham lesion) and thin spines (F(2, 12) = 13.40, p=0.0009; p = 0.001 vs. Sham lesion), but increased the number of thick spines (F(2, 12) = 59.6, p <0.0001; p <0.0001 vs. Control and vs. Sham lesion). DH-L had no effect on the number of bifurcated and unsorted spines of these neurons. The morphological classification of dendritic spines of the CA3 region (**Figure 5C**), a decrease of mushroom-like spines was observed (F(2, 12) = 61.78, p <0.0001; p < 0.0001 versus Control and Sham lesion) and thin spines (F(2, 12) = 10.63, p =0.0022; p = 0.0266 versus Sham lesion), but did increase the number of thick spines (F(2, 12) = 94.77, p < 0.0001; p <0.0001 versus control and sham lesion). DH-L also had no effect on the number of bifurcated and unsorted spines of these neurons.

Lastly, the morphological classification of dendritic spines of the DG region (**Figure 5D**), a decrease of mushroom-like spines was also observed in the (F(2, 12) = 48.18, p <0.0001; p < 0.0001 vs. control and sham lesion) and thin spines no significant differences were observed, but the number of thick spines increased (F(2, 12) = 61.16, p < 0.0001; p <0.0001 vs. Control and Sham lesion). DH-L had no effect on the number of bifurcated and unsorted spines of these neurons.

## 4 Discussion

In the present study, the VH-lesioned animals presented a significant decrease in the short- and long-term memory retention index compared to the VH-Sham and Control groups. The decrease in serum estrogen concentration in these animals may explain their results in the NORT test, since estrogens are a determining factor in the memory and learning process (46, 47). In ovariectomized female mice with global cerebral ischemia present a decrease in estrogen receptor proteins and aromatase expression in the hippocampus that affect their ability to discriminate tasks, such as learning and memory (48). Estrogen receptors Erα and ERβ are critical for memory preservation after cerebral ischemia in adult female rodents (49). Interestingly, analysis of locomotor activity showed increased hyperactivity in DH-injured animals compared with DH-Sham and Control groups. Locomotor activity to novel environment allows assessment of dopaminergic tone in the NAcc indirectly (32). Therefore, increased locomotor activity suggests hyperactivity of the mesolimbic pathway in DH-lesioned rats, altering the connectivity of the corticolimbic system, as well as mediating in part the effects of biologically significant stimuli on learning (50).

The observed decrease in short- and long-term memory in the Sham groups compared to the Control group may be attributed to alterations in the afferent and efferent projection fields of the hippocampus. These alterations could impact structures such as the septum, amygdala, cortex, and nucleus accumbens, likely as a consequence of the surgical procedure used to access the ventral or dorsal hippocampus (51, 52). Therefore, the differences observed between the hippocampal lesion groups and their respective Sham groups in the novel object recognition test (NORT) are more likely associated with the specific effects of the ibotenic acid-induced lesion (52, 53). Furthermore, the observed reduction in dendritic spine density in the Sham groups, along with an even more pronounced reduction in the hippocampal lesion groups, further supports this hypothesis.

In groups with ventral or dorsal hippocampal lesions induced by ibotenic acid, it is likely that mechanisms mediated by proinflammatory substances, which increase the activity of glial cells such as astrocytes and microglia. This increased glial activity is primarily driven by the activation of microglial mGluR5 receptors (54). Notably, this pathway may also trigger anti-inflammatory mechanisms that enhance cognitive function, as observed in both the prefrontal cortex and hippocampus (55).

Our results showed that the lesion performed in the VH or DH caused a delay in the age of vaginal opening and first vaginal estrus compared to animals with sham lesion and the control group. Interestingly, these animals also showed a significant decrease in the density of dendritic spines of CA1, CA3 and DG hippocampal pyramidal neurons compared to their respective control groups. The lesion in VH or DH probably blocked important sensory pathways that activate neuroendocrine mechanisms that regulate follicular growth and maturation, secretion of sex steroid hormones and gonadotropins, and thus fertility. It is transcendental that hippocampal lesions not only affected short- and long-term memory, but also provoked changes in reproductive parameters, in the concentration of steroid and gonadotropic hormones, in ovarian morphology and in the modulation of neuronal plasticity. Some studies provide evidence that allows us to consider the possibility that the hippocampus maintains bidirectional communication with the gonads (28, 29). It remains unclear whether this pathway is direct or mediated indirectly through the hypothalamus, amygdala, and cortical pathways (56–60). We have shown that sensory denervation causes a delay in puberty and in the age of first vaginal estrus (23, 24, 61). Puberty is an important period for the development of cognitive and signal integration functions and determines hippocampal function in adulthood (62). Estrogens stimulate the neuroendocrine mechanisms that initiate puberty (63, 64). Endocrine-disrupting compounds with estrogenic activity can delay puberty and exert detrimental effects on neurogenesis in the dentate gyrus, potentially leading to impaired learning and memory (65). There are few data evidencing the role of hippocampal involvement in the onset of puberty; however, estrogens may be a key factor as they regulate synaptic plasticity in the hippocampus of female rodents (66–69). In the ovariectomized rat, estrogen administration increases the density of dendritic spines in CA1 pyramidal cells of the hippocampus (68). Estrogen-induced plastic changes in CA1, CA3, and DG pyramidal neurons could contribute to the activation of neuroendocrine mechanisms that regulate the onset of puberty.

There is a bidirectional relationship between the hypothalamic-pituitary-gonadal (HPG) axis and the hippocampus, which, along with other structures of the limbic system, exhibits a high density of gonadotropin-releasing hormone (GnRH) receptors (70). Activation of these receptors in hippocampal pyramidal neurons within the CA1 and CA3 regions has been shown to enhance long-term neuronal excitability (71, 72).

It is possible that lesions in the ventral or dorsal hippocampus resulted in increased GABAergic tone and a reduction in excitatory inputs to the hypothalamus, which may have inhibited the secretion of GnRH (64). This effect, in conjunction with decreased estrogen secretion, could have contributed to the delayed onset of puberty. The impact may also be evident at the time of first vaginal estrus, as animals with hippocampal lesions exhibit reduced gonadotropin secretion. Supporting this hypothesis, during ovarian senescence, the loss of negative feedback from estrogen and inhibin leads to elevated GnRH concentrations, resulting in increased follicle-stimulating hormone (FSH) and luteinizing hormone (LH) levels (73). Notably, hippocampal pyramidal neurons, which are particularly vulnerable to Alzheimer’s disease, express LH receptors (74), and their activation has been associated with biochemical and cellular processes linked to neurodegeneration (72). Further research is required to determine whether the hippocampus can influence the pulsatility of GnRH secretion in the hypothalamus across different stages of life, beyond its established role during senescence.

Our results support the idea of a bidirectional connection between the hippocampus and the ovaries. This connection is advantageous for both components, the ovaries require neural and neuroendocrine signals, mediated in part by the hippocampus, for the control of follicular growth and the secretion of sex steroid hormones, in particular estrogens. These have receptors in the hippocampus to modulate its neuronal plasticity, the establishment of connections, neuronal survival and control of GABAergic tone (75–77). In support of the above, it has been demonstrated that in women, early bilateral salpingo-oophorectomy (BSO) causes a decrease in connectivity between both hippocampi and between the anterior and posterior part of the left hippocampus, decreasing associative memory (78). Women with BSO have an increased risk of developing Alzheimer’s disease and accelerated cognitive decline (79, 80). Sensory information sent from the ovaries to the CNS stimulates follicular growth in a stimulatory manner (23, 61). Any sensory information received by the hippocampus is processed by the entorhinal cortex, enters the hippocampus through two pathways: the trisynaptic loop from the dentate gyrus CA3 and CA1 and then back to the entorhinal cortex and the temporoammonic pathway, from the entorhinal cortex directly to CA1 (81). The CA1 region then sends axonal projections to the subiculum. Afferents from the subiculum travel through the corticohypothalamic tract, passing through the paraventricular nucleus (PVN) and the suprachiasmatic nucleus before terminating in the dorsomedial hypothalamus and mammillary bodies. Additionally, cells in the PVN project directly to the ventral subiculum and send some projections to hippocampal area CA1 (58). The hippocampus would translate excitatory inputs into inhibitory outputs, as well as encode a specific pattern to generate signals that regulate endocrine secretions (76). In this work, the ventral or dorsal lesion of the hippocampus could have modified this bidirectional communication, which would explain the increase in folliculogenesis and failures in steroidogenesis. This response has been seen in other studies that have induced local or peripheral denervation. The increase in follicles that we observed was mostly atretic, is understandable because the signals controlling the neural and neuroendocrine mechanisms that regulate hormone secretion are affected, for example, as is a lower secretion of progesterone and estradiol. Several studies on the involvement of ovarian nerves in controlling the secretion of sex steroid hormones (23, 24, 61, 82–84) and gonadotropins (61, 85, 86) support this hypothesis.

The significant reduction in the number of ovarian follicles and the lower secretion of sex steroid hormones observed in the sham animals compared to the control group reinforce the idea that the surgical procedure used to access the ventral or dorsal hippocampus affects afferent and efferent pathways. These pathways not only contribute to memory and learning processes (87, 88) but also play a role in the regulation of ovarian functions, including follicular growth and hormone secretion.

The increase in serum testosterone concentrations observed in the hippocampal lesion groups would also explain this increase in atretic follicles (89–93) as well as the total increase in follicles as an inducing effect of androgens on the onset of follicular growth (90, 93, 94). It would also account for the increased locomotion observed in DH-lesioned animals by an increase in hippocampal excitability, as has been shown in the hyperandrogenism model with the female PCOS rat (95). The decrease in serum FSH concentrations observed in the hippocampal lesion groups would be another factor contributing to the increase in atretic follicles in hippocampal lesion animals (92, 96, 97). This diminished response in ovarian functions, gonadotropin secretion and alterations in neural communication between the hippocampus and ovaries would explain a greater difficulty for females with hippocampal lesion, more dorsal than ventral, to become pregnant.

The decrease of estrogens in the group of animals with hippocampal lesion would explain the decrease in the density of dendritic spines, especially of the mushroom type. Estrogens are known to regulate reproductive behavior, neurodevelopment, plasticity and hippocampal function (68, 98). Woolley and collaborators (69) demonstrated the effect of estradiol and progesterone in ovariectomized rats on the density of dendritic spines in CA1 pyramidal cells of the hippocampus of female rats, where they observed an increase in dendritic density after twenty-four hours following the administration of estradiol, On the other hand, they showed that progesterone has a biphasic effect on spine density, i.e., progesterone administration after estradiol causes an initial increase, but then causes a much sharper decrease.

These results are similar to those previously found by several groups (99–101) who suggest natural fluctuations in dendritic spine density and estrogen receptor expression during the estrous cycle. It cannot be ruled out that the changes observed in the dendritic morphology of CA1, CA3 and DG pyramidal neurons in animals with lesions in VH and DH are associated with the effect of an interruption in communication in the ovary-hypothalamus-hippocampus neural pathway that stimulated a delay in puberty and the aforementioned changes in follicular growth. It is possible that the lesion in the ventral or dorsal hippocampus may have caused intact hippocampal areas to have less excitatory sensory stimulus from the ovaries. In support of this, the plasticity of dendritic spines may be modified by several factors, such as actin that is regulated by Rho family GTPases and AMPA receptors that promote dendritic spine formation by increasing Ca2+ levels in hippocampal neurons. Estrogen-regulated NMDA receptors have also been associated with an increase in the size and density of dendritic spines of hippocampal pyramidal neurons (102–105). In addition to changes in dendritic morphology potentially induced by decreased estradiol levels, these alterations may also result from reduced levels of neurotrophic factors, such as brain-derived neurotrophic factor (BDNF) and inducible nerve growth factor B (NGFB), which are observed in animals with hippocampal injury (106–108). These factors may contribute to functional alterations in the hippocampus and its neural circuits, affecting communication with other central nervous system structures (109, 110). This supports our hypothesis that hippocampal lesions lead to disruptions in bidirectional communication between the hippocampus and ovaries.

Kolb’s group (111) suggest that changes in neuronal morphology may be completely independent. Hippocampal damage may have important implications not only for the function of the hippocampus itself such as memory and learning, but also for understanding the behavior of the individual. In addition, the involvement and influence of ovarian hormones may have particularly important implications for differential synaptic plasticity at different times of the menstrual cycle. Estrogens are known to promote some spines to change from a thin filopodia-like structure to spines with larger, well-defined, mushroom-like necks and heads (112–114). Consistent with the increase in thin and stubby spines observed and a decrease in mushroom-like spines and the decrease in serum estradiol concentrations observed in the VH-L and DH-L groups in our study.

The existence of a hippocampus-pituitary-gonads axis has been proposed, whose function would be to ensure hippocampal plasticity in the adult brain through the regulation of steroid secretion of ovarian and neural origin (114–116). According to our results, the neuroendocrine mechanisms that regulate reproduction and cognitive processes are mutually dependent, and it would be necessary to keep this in mind from now on for a holistic analysis of reproductive and cognitive problems. Probably, the existence of a Hippocampus-Hypothalamus-Hypothalamus-Pituitary-Gonads (H-HPG) axis would have to be considered.

In summary, these results show, for the first time, that the hippocampus participates in a stimulatory manner in the regulation of ovarian function, through a neural connection with the gonads. This leads to an integrative pathway between learning processes and the neuroendocrine mechanisms that regulate reproduction. Further studies are required to anatomically delineate the neural pathway between the ovaries and the hippocampus.

## Data Availability

The raw data supporting the conclusions of this article will be made available by the authors, without undue reservation.
